# Supplementary material for: Differential regulation of hepatic physiology and injury by the TAM receptors Axl and Mer
Source: Life Sci Alliance. 2020 Jun 22;3(8):e202000694. doi: 10.26508/lsa.202000694 (PMC7335405; doi:10.26508/lsa.202000694)
Supplement: Supplementary file 1 [file LSA-2020-00694_TableS1.docx]

Supplementary Table S1.

| **Target mRNA** |  | **Primer sequence** |
| --- | --- | --- |
| Ppia (CypA) | Forward primer | GCCGATGACGAGCCCTT |
|  | Reverse primer | AAGTCACCACCCTGGCACA |
| Rplp0 (36B4) | Forward primer | CTCTCGCTTTCTGGAGGGTG |
|  | Reverse primer | ACGCGCTTGTACCCATTGAT |
| Tnf | Forward primer | GCCACCACGCTCTTCTGTCT |
|  | Reverse primer | CAGCTGCTCCTCCACTTGGT |
| Cd5l (AIM) | Forward primer | TTTGTTGGATCGTGTTTTTCAGA |
|  | Reverse primer | CTTCACAGCGGTGGGCA |
| Siglec1 (CD169) | Forward primer | CTTGGGTCAGCCAACAGTTC |
|  | Reverse primer | GGTGATGGTGAAACCTGGAC |
| Il10 | Forward primer | TGAAGACCCTCAGGATGCG |
|  | Reverse primer | TTCACCTGCTCCACTGCCTT |
| Il1b | Forward primer | CCTCTCCAGCCAAGCTTCC |
|  | Reverse primer | CTCATCAGGACAGCCCAGGT |
| Marco | Forward primer | GGCACCAAGGGAGACAAA |
|  | Reverse primer | TCCCTTCATGCCCATGTC |
| Ccl5 (RANTES) | Forward primer | GTGCCCACGTCAAGGAGTAT |
|  | Reverse primer | CCCACTTCTTCTCTGGGTTG |
| Ifna4 | Forward primer | CCCACAGCCCAGAGAGTGAC |
|  | Reverse primer | GCCCTCTTGTTCCCGAGGT |
| Ifnb | Forward primer | CCAGCCTGGCTTCCATCAT |
|  | Reverse primer | TGTGGTGGAGAAGCACAGCA |
| Il6 | Forward primer | AGACAAAGCCAGAGTCCTTCAGA |
|  | Reverse primer | GCCACTCCTTCTGTGACTCCA |
| Ccl2 (MCP1) | Forward primer | TGCTGACCCCAAGAAGGAATG |
|  | Reverse primer | GCTGAAGACCTTAGGGCAGAT |
| MMP12 | Forward primer | TTTGGATTATTGGAATGCTGC |
|  | Reverse primer | ATGAGGCAGAAACGTGGACT |
